# Substrate sustained release-based high efficacy biosynthesis of GABA by *Lactobacillus brevis* NCL912

**DOI:** 10.1186/s12934-018-0919-6

**Published:** 2018-05-19

**Authors:** Qiong Wang, Xiaohua Liu, Jinheng Fu, Shuixing Wang, Yuanhong Chen, Kunpeng Chang, Haixing Li

**Affiliations:** 1 State Key Laboratory of Food Science and Technology, Nanchang University, Nanchang, 330047 People’s Republic of China; 2 Sino-German Joint Research Institute, Nanchang University, Nanchang, 330047 People’s Republic of China

**Keywords:** *Lactobacillus brevis* NCL912, Sustained release of l-glutamic acid, pH self-control of l-glutamic acid, Gamma-aminobutyric acid

## Abstract

**Background:**

Gamma-aminobutyric acid (GABA) plays a significant role in the food and drug industries. Our previous study established an efficient fed-batch fermentation process for *Lactobacillus brevis* NCL912 production of GABA from monosodium l-glutamate; however, monosodium l-glutamate may not be an ideal substrate, as it can result in the rapid increase of pH due to decarboxylation. Thus, in this study, l-glutamic acid was proposed as a substrate. To evaluate its potential, key components of the fermentation medium affecting GABA synthesis were re-screened and re-optimized to enhance GABA production from *L. brevis* NCL912.

**Results:**

The initial fermentation medium (pH 3.3) used for optimization was: 50 g/L glucose, 25 g/L yeast extract, 10 mg/L manganese sulfate (MnSO_4_·H_2_O), 2 g/L Tween-80, and 220 g/L l-glutamic acid. Glucose, a nitrogen source, magnesium, and Tween-80 had notable effects on GABA production from the l-glutamic acid-based process; other factors showed no or marginal effects. The optimized levels of the four key components in the fermentation medium were 25 g/L glucose, 25 g/L yeast extract FM408, 25 mg/L MnSO_4_·H_2_O, and 2 g/L Tween-80. A simple and efficient fermentation process for the bioconversion of GABA by *L. brevis* NCL912 was subsequently developed in a 10 L fermenter as follows: fermentation medium, 5 L; glutamic acid, 295 g/L; inoculum, 10% (v/v); incubation temperature, 32 °C; and agitation, 100 rpm. After 48 h of fermentation, the final GABA concentration increased up to 205.8 ± 8.0 g/L.

**Conclusions:**

l-Glutamic acid was superior to monosodium l-glutamate as a substrate in the bioproduction of GABA. Thus, a high efficacy bioprocess with 205 g/L GABA for *L. brevis* NCL912 was established. This strategy may provide an alternative for increasing the bioconversion of GABA.

## Background

Gamma-aminobutyric acid (GABA) is a non-protein amino acid that is widely distributed in nature, and functions as a major inhibitory neurotransmitter in the mammalian central nervous system [1]. GABA possesses numerous well-known physiological properties, such as tranquilizing [2], anti-anxiety [3], diuretic, hypotensive [4] and diabetes reversal [5] effects. Therefore, it is commonly used in the food and pharmaceutical industries. Many GABA-enriched functional products, such as fermented vegetables [6], milk [7, 8], seafood [9, 10] and soybean [11], have been produced and exhibit various health-promoting effects. Recently, GABA has garnered much attention for use as a precursor for the synthesis of 2-pyrrolidone and nylon 4, which has increased its possible applications in the chemical industry [12, 13].

There is an increasing demand for GABA in the drug and food industries, and in industrial biotechnology. Thus, much attention has been paid to the large-scale bioconversion of GABA by microbes. GABA production by lactic acid bacteria (LAB) has been particularly studied, as LAB are generally recognized as safe organisms [14–16]. A large number of LAB strains are able to synthesize GABA and are distributed in species including *Lactobacillus brevis* [8], *L. paracasei* [17], *Lactococcus lactis* [18], and *L. plantarum* [19], some of which have potential industrial applications due to their high GABA-producing abilities [1, 20–22]. Tamura et al. [22] developed a fed-batch fermentation method for the efficient biotransformation of monosodium l-glutamate (MSG) by *Enterococcus avium* G-15, in which the final GABA content reached 115.7 g/L after 72 h. Shi et al. [23] established a cell biotransformation process for converting a mixed substrate (80 g/L l-glutamic acid plus 240 g/L MSG) into GABA by high levels of rinsed *L. brevis* TCCC13007 resting cells, generating 201 g/L GABA. MSG has been widely applied to GABA fermentation processes [22–24], possibly due to its good solubility.

Acidic conditions are essential for the decarboxylation of glutamate catalyzed by glutamic acid decarboxylase (GAD). A glutamate molecule is decarboxylated into GABA by GAD, with the concomitant release of carbon dioxide and hydrogen (H^+^) consumption, resulting in a pH increase [25]; thus, GAD enzymatic activity is gradually inhibited with the decarboxylation reaction. To maintain the acidic pH needed for GAD to exert its enzymatic activity, the transformation system has to be continuously supplemented with an acidic reagent [1, 22, 26]. In our previous study, a simple fed-batch fermentation process was developed for the efficient synthesis of GABA by *L. brevis* NCL912. During the fermentation process, large sulfuric acid (H_2_SO_4_) concentrations were used to counterbalance the increase in pH. However, the added sulfate (SO_4_^2−^) was harmful to the cells and thus inhibited the synthesis of GABA [26].

There have been no reports on the use of l-glutamic acid as a substrate for GABA production. Compared to MSG, l-glutamic acid has much lower solubility, and the substitution of H^+^ for a sodium ion (Na^+^) on the α-carboxyl group may alleviate the inhibitory effects caused by MSG, thereby improving GABA production. In this context, the culture parameters for *L. brevis* NCL912 were re-optimized based on l-glutamic acid. Under optimal conditions (fermentation medium containing 295 g/L l-glutamic acid, 10% [v/v] inoculum, incubation temperature of 32 °C, agitation of 100 rpm), the concentration of GABA in the fermentation broth increased by almost twofold, reaching 205 g/L at 48 h. Thus, the application of l-glutamic acid as a substrate in fed-batch processes may be useful for the biosynthesis of GABA by microbes.

## Methods

### Materials

Nitrogen sources including yeast extracts FM408, FM405, FM803, FM818, FM828, and FM888; bovine heart extract 81001494; beef extract 81001536; bovine liver extract 81001539; tryptone FP318; soy peotones FP410 and FP328; and yeast peptone FP103 were purchased from Angel Yeast Co., Ltd. (Yichang, China). Other nitrogen sources including yeast extracts 02-03A, 02-12A, 02-33, 02-12C, 02-24, 02-41, and 02-49; egg albumin extract 02-57; gelatin peptone 02-48; bacterial peptone 02-31C; tryptones 02-71, 02-04, and 02-04C; casein peptones 02-11A, 02-11B, and 02-34; proteose peptone 02-27; fish peptone 02-40; beef peptone 02-07; soy peptones 02-19 and 02-31; polypeptone 02-02B; and lactoalbumin hydrolysate 02-13 were purchased from Shuangxuan Microbe Culture Medium Products Plant (Beijing, China). Other reagents were of analytical grade or biochemically pure.

3,5-Dinitrosalicylic acid (DNS) reagent was made by mixing solution A and B. Solution A was prepared by dissolving 6.9 g crystalline phenol in 15.2 mL 10% sodium hydroxide (NaOH), diluting to 69 mL, and then adding 6.9 g sodium hydrogen sulfite (NaHSO_3_). Solution B was prepared by adding 255 g sodium potassium tartrate (C_4_H_4_O_6_KNa·4H_2_O) into 300 mL 10% NaOH, which then was mixed with 880 mL 1% DNS. The prepared DNS reagent was available after being stored in a brown bottle for 7–10 days at room temperature [27].

### Bacterial strain and media

*Lactobacillus brevis* NCL912 (=CCTCCM208054), a high GABA producer, was isolated from Chinese traditional paocai [28]. Seed medium at a pH of 5 contained: 50 g/L glucose, 12.5 g/L yeast extract, 12.5 g/L soy peptone, 10 mg/L manganese sulfate (MnSO_4_·H_2_O), 28 g/L MSG, and 2 g/L Tween-80. The initial fermentation medium was composed of 50 g/L glucose, 25 g/L yeast extract, 10 mg/L MnSO_4_·H_2_O, 220 g/L glutamic acid, and 2 g/L Tween-80. The nitrogen source, substrate, and other components were separately autoclaved at 121 °C for 30 min, and mixed prior to inoculation [26, 29].

### Culture conditions

After cultivation for approximately 10 h at 32 °C and 100 rpm in the seed medium, the absorbance at 600 nm (*A*_600_) of NCL912 seed broth was 4–6 and could be used as the inoculum. Fermentation optimization experiments were performed in 250 mL conical flasks in a medium volume of 100 mL without shaking. Unless otherwise specified, 220 g/L glutamic acid powder was added to each conical flask, and the inoculum volume was 10 mL. To exclude any GABA synthesis promoting roles in Mn^2+^ or Tween-80-free experiments, these two residues in seed culture were removed prior to inoculation as follows: the seed broth was centrifuged to recover the cells, after which the cells were suspended in an equal volume of 0.9% sodium chloride (NaCl) after being washed once. The suspension was used as the inoculum. The inoculated flasks were kept at 32 °C, and samples were taken every 12 h to measure GABA content. Batch fermentation was performed in a 10 L fermenter to confirm the optimized parameters in flask culture. The batch fermentation conditions were: fermentation medium load, 5 L; inoculum volume, 10% (v/v); incubation temperature, 32 °C; agitation speed, 100 rpm; l-glutamic acid, 295 g/L; and fermentation period, 48 h. Samples were taken at intervals of 12 h.

### Analytical procedures

Cell growth was monitored by measuring the absorbance value at 600 nm (*A*_*600*_) on a UV–vis spectrophotometer (MAPADA Instrument; Shanghai, China). GABA contents were quantitatively determined using pre-staining paper chromatography coupled with vis spectrophotometry [30]. A sample volume of 2 μL (diluted if necessary) was spotted onto chromatography paper. The paper was developed at 30 °C with n-butanol-acetic acid–water (5:3:2) containing 12 g/L ninhydrin. After development, the paper was directly dried for color yield at 70 °C for 80 min. Then the GABA spots were cut out from the paper and extracted with 5 mL of 75% alcohol (v/v):0.6% cupric sulfate (w/v) (38:2, v/v) at 40 °C and 50 rpm for 60 min. The absorption was read in the spectrophotometer at 512 nm. Glucose concentrations were quantified by the DNS reagent method [27]. In brief, 1 mL 50-fold diluted samples, 1 mL distilled water, and 1.5 mL DNS reagent were mixed and cooled with cold running water immediately after being heated for 5 min in a boiling water bath. Then 21.5 mL distilled water was added and mixed. *A*_520_ values of the mixtures were obtained in the spectrophotometer.

## Results and discussion

### l-Glutamic acid is a promising alternative to MSG

Excessive MSG reportedly inhibits GABA formation; however, the underlying mechanism remains to be clarified. Nevertheless synchronous cell growth inhibition has been observed [17, 22, 26, 31], implying that excessive MSG may act as an impediment to GABA production by reducing cell mass. Fed-batch processes have been employed to lower the adverse effects of excess MSG [22, 26], but they also contribute to the operation complexity and cost of fermentation. In addition, when MSG is used as the substrate, a large amount of acid solution is consumed to offset the pH increase arising from both the decarboxylation reaction and MSG itself (neutral pH). In our previous fed-batch process for NCL912, continuous H_2_SO_4_ flow and MSG supplementation significantly contributed to the complexity of the process and cost of fermentation. In addition, the harshness of the environment was aggravated by the additional Na_2_SO_4_, which was harmful to the strain and inhibited GABA synthesis. Moreover, the produced by-product Na_2_SO_4_ increased the difficulty of purifying the end product [26, 32]. Compared to MSG, l-glutamic acid has much lower solubility, and the substitution of H^+^ for a Na^2+^ on the α-carboxyl group confers l-glutamic acid the properties of sustained release and pH buffering. Therefore, glutamic acid may have the advantage of needing to only be added one time prior to cultivation due to its sustained release. In addition, extra inorganic acid would not be needed, as glutamic acid can self-maintain an acidic pH. These key characteristics of glutamic acid may allow efficient GABA production by reducing the inhibition of extra by-products. In this study, the formulation of the culture medium was re-optimized for the synthesis of GABA by *L. brevis* NCL912. Subsequently, l-glutamic acid biotransformation was conducted in a 10 L fermenter under optimized flask parameters. The results showed that l-glutamic acid contributed to the higher efficacy of GABA biosynthesis.

### Effects of glucose on GABA production

**Fig. 1:**
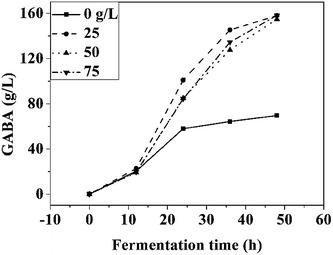
Effects of glucose on GABA production. Data are expressed as the mean of three independent experiments

High concentrations of glucose aggravate the harshness of the environment due to the fact that *L. brevis* NCL912 metabolizes glucose into low molecular weight organic acids. Therefore, in glutamic acid-based fermentation, high concentrations of glucose may have adverse effects on GABA synthesis. In the MSG-based process, MSG decarboxylation alkalizes the culture fluid, and in this condition, organic acids produced from glucose may have a positive effect on decarboxylation. Thus a lower concentration of glucose may be required for glutamic acid-based fermentation compared to MSG-based fermentation. However, the mechanisms underlying this difference remain unknown. Based on our former work and preliminary experiments, the following initial fermentation medium (pH 3.3) was used: 50 g/L glucose, 25 g/L yeast powder, 10 mg/L MnSO_4_·H_2_O, 2 g/L Tween-80, and 220 g/L l-glutamic acid [26, 29]. As shown in Fig. 1, glucose had a significant effect on GABA production. However, the amount of GABA produced was similar with 25 g/L glucose or higher glucose concentrations, indicating that higher glucose concentrations (> 25 g/L) did not facilitate GABA synthesis. Thus, the optimal concentration of glucose for the current process was 25 g/L, which was much lower than that used for MSG-based fed-batch fermentation [26]. The possible reason for this discrepancy may be due to the type of substrate used in the reaction.

### Effects of nitrogen sources on GABA synthesis

**Fig. 2:**
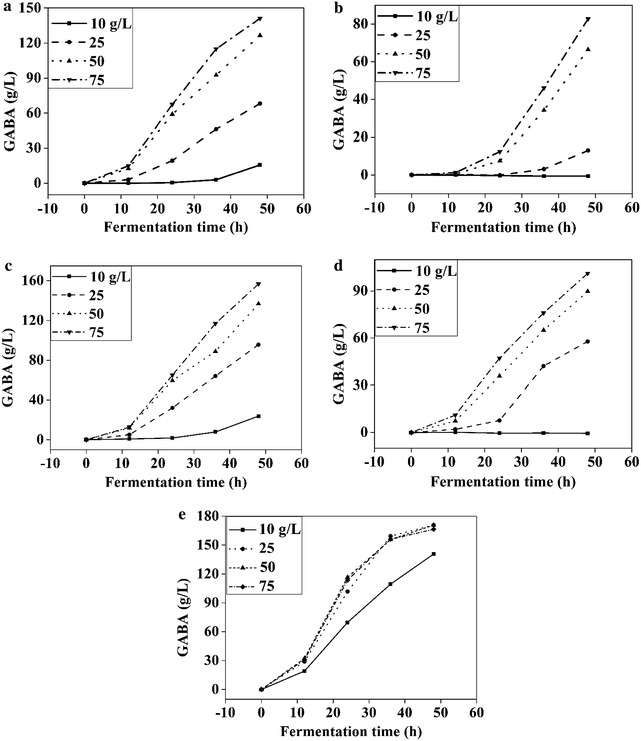
Effects of nitrogen sources on GABA production. **a** Yeast extract 02-12C; **b** yeast extract 02-12A; **c** yeast extract FM405; **d** soy peptone FP410; **e** yeast extract FM408. Data are expressed as the mean of three independent experiments

A total of 36 nitrogen sources were individually tested for their effects on GABA production by *L. brevis* NCL912, of which 31 showed relative low GABA production ability as substantial glutamic acid powder remained in the medium even after 96 h (data not shown). The remaining five nitrogen sources with relatively high GABA production were further analyzed in a 10–75 g/L concentration range to determine the best nitrogen source and optimal concentration. As shown in Fig. 2, the yeast extract FM408 was optimal for GABA synthesis. At a relatively low concentration of FM408 (< 25 g/L), GABA formation increased with increasing FM408 concentration, but when the concentration exceeded 25 g/L, the increase in GABA production was almost negligible. Therefore, 25 g/L FM408 was selected for subsequent experiments. Previously, nitrogen sources from Shuangxuan were tested, of which soy peptone 02-19 was shown to be the optimal nitrogen source for GABA production from *L. brevis* NCL912 [29]. In the current study, 36 nitrogen sources obtained from Shuangxuan and Angel were investigated in parallel for their abilities to convert glutamic acid into GABA by *L. brevis* NCL912. The yeast extract FM408 was found to be the best nitrogen source for the purpose of this study.

### Effects of Tween-80 on GABA yield

**Fig. 3:**
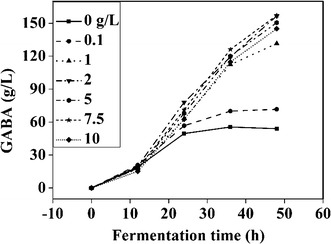
Effects of Tween-80 on GABA production. Data are expressed as the mean of three independent experiments

The effects of Tween-80 on GABA synthesis are presented in Fig. 3. Tween-80 had distinct effects on GABA production, as GABA production increased with increasing Tween-80 concentration from 0 to 2 g/L; however, there was no significant increase in GABA production at a Tween-80 concentration higher than 2 g/L. Thus, 2 g/L Tween-80 was sufficient for the maximum GABA production. Tween-80 is a growth stimulator for most LAB [1, 18, 33]. Therefore, Tween-80 probably elevated GABA production by promoting *L. brevis* NCL912 cell growth, as little *L. brevis* NCL912 grew concomitantly with little GABA production in the absence of Tween-80. In addition, as a surfactant, Tween-80 can improve cell membrane permeability; thus, it may have promoted GABA synthesis by increasing the efficiency of the glutamate-GABA antiporter.

### Effects of amino acids on GABA synthesis

It was presumed that amino acids may contribute to GABA production, because they serve as energy sources and provide structural support in organisms. Therefore, 21 proteinogenic amino acids were evaluated for their effects on GABA production. Interestingly, the results showed that none of them promoted GABA production (data not shown), possibly due to the fact that the yeast powder FM408 had sufficient levels of essential amino acids for GABA production by *L. brevis* NCL912.

### Effects of other compounds on GABA production

**Fig. 4:**
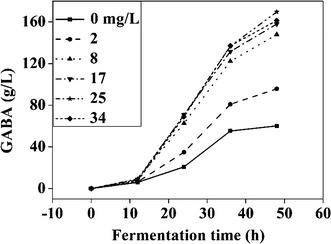
Effects of Mn^2+^ on GABA production. Data are expressed as the mean of three independent experiments

A total of 20 potential compounds including MnSO_4_·H_2_O, KNO_3_, KH_2_PO_4_, NaCl, CaCl_2_, FeCl_3_·6H_2_O, MgSO_4_·7H_2_O, CuSO_4_·5H_2_O, NH_4_NO_3_, Al(NO_3_)_3_·9H_2_O, VB_1_, VB_6_, VB_7_, VB_12_, NAD, NADPNa_2_, Zn(CH_3_COO)_2_·2H_2_O, sodium citrate, linoleic acid, and mannitol were evaluated for their effects on GABA production by *L. brevis* NCL912. The results indicated that Mn^2+^ could significantly improve GABA production, whereas the other compounds exhibited no or only marginal stimulatory effects indicated by little consumption of glutamic acid powder even after 96 h (data not shown), consistent with a previous report in which MSG was used as the substrate [29]. It was presumed that Mn^2+^ may promote GABA production by increasing GAD enzymatic activity and transportation efficiency of the glutamate-GABA antiporter; however, confirmatory studies are warranted. Figure 4 shows the effects of Mn^2+^ concentration on GABA production. GABA production increased with increasing MnSO_4_·H_2_O concentration from 0 to 25 mg/L, but higher MnSO_4_·H_2_O concentrations did not markedly increase GABA production. Thus, a concentration of 25 mg/L MnSO_4_·H_2_O was selected for subsequent experiments.

### Efficient batch fermentation for GABA

**Fig. 5:**
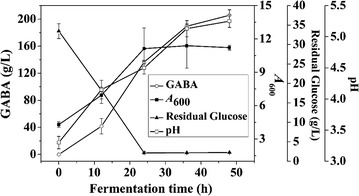
Time courses of GABA production, cell growth, pH, and residual glucose in batch fermentation. Bars represent the standard error of the mean (n = 3)

A fermentation medium (pH 3.3) based on the abovementioned optimized parameters comprised the following: 25 g/L glucose, 25 g/L yeast extract FM408, 25 mg/L MnSO_4_·H_2_O, and 2 g/L Tween-80. The culture was incubated in a 10 L fermenter with 5 L fresh medium. A concentration of 295 g/L l-glutamic acid and 500 mL seed culture were added to the fermenter prior to fermentation. Figure 5 shows that cell growth occurred immediately after inoculation and biomass rapidly increased after the first 12 h; by 36 h, cell growth was almost inhibited. Accordingly, GABA was rapidly produced from 0 to 36 h, and to a lesser extent from 36 to 48 h. After exceeding 200 g/L at 48 h, the synthesis of GABA was severely inhibited; thus the optimal fermentation period was determined to be 48 h. The final GABA yield at 48 h was 205.8 ± 8.0 g/L, which was 102% higher than the yield obtained from a MSG-based fed-batch [26]. The initial pH of the inoculated fermentation medium saturated by l-glutamic acid was approximate 3.3, which is harsh to numerous microbes. The glutamate-dependent GAD system in *L. brevis* NCL912, however, confers resistance to low pH [26], making *L. brevis* NCL912 cells grow well even in an extremely low pH environment. The pH was gradually increased from 3.3 to 5.3 during the process, a pH range that favors glutamate decarboxylation by GAD [26, 34, 35]. The same molar mass of MSG and glutamic acid are similar in cost, but the proposed bioprocess was simpler, more economical, and more efficient than MSG-based fermentation. Gao and colleagues [23] exploited a cell bioconversion for the efficient production of GABA, and the final GABA content was up to 201 g/L. However, in that process, highly concentrated rinsed *L. brevis* TCCC13007 resting cells (50 g/L) were employed to bioconvert glutamate [23], and GABA content in fermentation of *L. brevis* TCCC13007 was only 61 g/L [36]. Similarly, although Zhao et al. [37] obtained 129 g/L GABA with the combination of fermentation and cell bioconversion of *L. buchneri* WPZ001, fermentation alone yielded only 70 g/L GABA at 48 h. In addition, extra operations and reagents were needed for those processes [36, 37]. Comprehensively, our current bioprocess developed for *L. brevis* NCL912 was much more efficient than previous ones used in other LAB for GABA production. The difference in GABA production by *L. brevis* NCL912 using glutamic acid- and MSG-based processes showed that using glutamic acid as the substrate led to greater GABA production. Although glutamic acid lowers the initial pH of media, the proposed glutamic acid-based strategy may be applied to other GAD-containing microbes due to the fact that the major role of the GAD system is to impart cellular acid resistance [34, 38–41]. Thus, it is reasonable to imagine that a glutamic acid-based process may enhance the GABA production from some LAB strains including *L. brevis* TCCC13007 and *L. buchneri* WPZ001 [36, 37].

## Conclusions

Due to benefits such as sustained release and self-maintaining acidic pH, l-glutamic acid instead of MSG was utilized as a substrate for GABA synthesis by *L. brevis* NCL912. The fermentation medium was re-optimized due to this change in substrate. Subsequently, high efficacy batch fermentation was developed, which yielded 205.8 ± 8.0 g/L GABA at 48 h, which was approximately double the yield obtained from the original fermentation process using MSG. This strategy of altering the substrate may be applied to various microbial GABA processes, thereby opening up avenues for the optimization of bioprocesses that maximize GABA yield.

## References

[1] Li H, Cao Y (2010). Lactic acid bacterial cell factories for gamma-aminobutyric acid. Amino Acids.

[2] Wong T, Guin C, Bottiglieri T, Snead OC (2003). GABA, gamma-hydroxybutyric acid, and neurological disease. Ann Neurol.

[3] Yoon YE, Kuppusamy S, Cho KM, Kim PJ, Kwack YB, Lee YB (2017). Influence of cold stress on contents of soluble sugars, vitamin C and free amino acids including gamma-aminobutyric acid (GABA) in spinach (*Spinacia oleracea*). Food Chem.

[4] Matsuo A, Sato K, Park EY, Nakamura Y, Ohtsuki K (2012). Control of amylase and protease activities in a phytase preparation by ampholyte-free preparative isoelectric focusing for unrefined cereal-containing bread. J Funct Foods.

[5] Marques TM, Patterson E, Wall R, O’Sullivan O, Fitzgerald GF, Cotter PD, Dinan TG, Cryan JF, Ross RP, Stanton C (2016). Influence of GABA and GABA-producing *Lactobacillus brevis* DPC6108 on the development of diabetes in a streptozotocin rat model. Benef Microbes.

[6] Bonatsou S, Iliopoulos V, Mallouchos A, Gogou E, Oikonomopoulou V, Krokida M, Taoukis P, Panagou EZ (2017). Effect of osmotic dehydration of olives as pre-fermentation treatment and partial substitution of sodium chloride by monosodium glutamate in the fermentation profile of Kalamata natural black olives. Food Microbiol.

[7] Li W, Wei M, Wu J, Rui X, Dong M (2016). Novel fermented chickpea milk with enhanced level of gamma-aminobutyric acid and neuroprotective effect on PC12 cells. PeerJ.

[8] Wu Q, Law YS, Shah NP (2015). Dairy *Streptococcus thermophilus* improves cell viability of *Lactobacillus brevis* NPS-QW-145 and its gamma-aminobutyric acid biosynthesis ability in milk. Sci Rep.

[9] Sanchart C, Rattanaporn O, Haltrich D, Phukpattaranont P, Maneerat S (2017). Enhancement of gamma-aminobutyric acid (GABA) levels using an autochthonous *Lactobacillus futsaii* CS3 as starter culture in Thai fermented shrimp (*Kung*-*Som*). World J Microbiol Biotechnol.

[10] Lee KW, Shim JM, Yao Z, Kim JA, Kim HJ, Kim JH (2017). Characterization of a glutamate decarboxylase (GAD) from *Enterococcus avium* M5 isolated from jeotgal, a Korean fermented seafood. J Microbiol Biotechnol.

[11] Jang EK, Kim NY, Ahn HJ, Ji GE (2015). Gamma-aminobutyric acid (GABA) production and angiotensin-I converting enzyme (ACE) inhibitory activity of fermented soybean containing sea tangle by the co-culture of *Lactobacillus brevis* with *Aspergillus oryzae*. J Microbiol Biotechnol.

[12] Grewal J, Khare SK (2017). 2-Pyrrolidone synthesis from gamma-aminobutyric acid produced by *Lactobacillus brevis* under solid-state fermentation utilizing toxic deoiled cottonseed cake. Bioprocess Biosyst Eng.

[13] Choi JW, Yim SS, Lee SH, Kang TJ, Park SJ, Jeong KJ (2015). Enhanced production of gamma-aminobutyrate (GABA) in recombinant *Corynebacterium glutamicum* by expressing glutamate decarboxylase active in expanded pH range. Microb Cell Fact.

[14] Lee NK, Paik HD (2017). Bioconversion using lactic acid bacteria: ginsenosides, GABA, and phenolic compounds. J Microbiol Biotechnol.

[15] Kok J, Johansen E, Kleerebezem M, Teusink B (2014). Lactic acid bacteria: embarking on 30 more years of research. Microb Cell Fact.

[16] Garcia-Fruitos E (2012). Lactic acid bacteria: a promising alternative for recombinant protein production. Microb Cell Fact.

[17] Komatsuzaki N, Shima J, Kawamoto S, Momose H, Kimura T (2005). Production of gamma-aminobutyric acid (GABA) by *Lactobacillus paracasei* isolated from traditional fermented foods. Food Microbiol.

[18] Siragusa S, De Angelis M, Di Cagno R, Rizzello CG, Coda R, Gobbetti M (2007). Synthesis of gamma-aminobutyric acid by lactic acid bacteria isolated from a variety of Italian cheeses. Appl Environ Microbiol.

[19] Tajabadi N, Ebrahimpour A, Baradaran A, Rahim RA, Mahyudin NA, Manap MY, Bakar FA, Saari N (2015). Optimization of gamma-aminobutyric acid production by *Lactobacillus plantarum* Taj-Apis362 from honeybees. Molecules.

[20] Lyu C, Hu S, Huang J, Luo M, Lu T, Mei L, Yao S (2016). Contribution of the activated catalase to oxidative stress resistance and gamma-aminobutyric acid production in *Lactobacillus brevis*. Int J Food Microbiol.

[21] Yang T, Rao Z, Kimani BG, Xu M, Zhang X, Yang ST (2015). Two-step production of gamma-aminobutyric acid from cassava powder using *Corynebacterium glutamicum* and *Lactobacillus plantarum*. J Ind Microbiol Biotechnol.

[22] Tamura T, Noda M, Ozaki M, Maruyama M, Matoba Y, Kumagai T, Sugiyama M (2010). Establishment of an efficient fermentation system of gamma-aminobutyric acid by a lactic acid bacterium, *Enterococcus avium* G-15, isolated from carrot leaves. Biol Pharm Bull.

[23] Shi X, Chang C, Ma S, Cheng Y, Zhang J, Gao Q (2017). Efficient bioconversion of L-glutamate to gamma-aminobutyric acid by *Lactobacillus brevis* resting cells. J Ind Microbiol Biotechnol.

[24] Lim HS, Cha IT, Roh SW, Shin HH, Seo MJ (2017). Enhanced production of gamma-aminobutyric acid by optimizing culture conditions of *Lactobacillus brevis* HYE1 isolated from kimchi, a Korean fermented food. J Microbiol Biotechnol.

[25] Cotter PD, Hill C (2003). Surviving the acid test: responses of gram-positive bacteria to low pH. Microbiol Mol Biol Rev.

[26] Li H, Qiu T, Huang G, Cao Y (2010). Production of gamma-aminobutyric acid by *Lactobacillus brevis* NCL912 using fed-batch fermentation. Microb Cell Fact.

[27] Department of Biology, Perking University (1991). Biochemical experiment guide.

[28] Li H, Gao D, Cao Y, Xu H (2008). A high γ-aminobutyric acid-producing *Lactobacillus brevis* isolated from Chinese traditional paocai. Ann Microbiol.

[29] Li H, Qiu T, Gao D, Cao Y (2010). Medium optimization for production of gamma-aminobutyric acid by *Lactobacillus brevis* NCL912. Amino Acids.

[30] Li H, Qiu T, Cao Y, Yang J, Huang Z (2009). Pre-staining paper chromatography method for quantification of gamma-aminobutyric acid. J Chromatogr A.

[31] Binh TT, Ju WT, Jung WJ, Park RD (2014). Optimization of gamma-amino butyric acid production in a newly isolated *Lactobacillus brevis*. Biotechnol Lett.

[32] Li H, Qiu T, Chen Y, Cao Y (2011). Separation of gamma-aminobutyric acid from fermented broth. J Ind Microbiol Biotechnol.

[33] Yunes RA, Poluektova EU, Dyachkova MS, Klimina KM, Kovtun AS, Averina OV, Orlova VS, Danilenko VN (2016). GABA production and structure of *gadB/gadC* genes in *Lactobacillus* and *Bifidobacterium* strains from human microbiota. Anaerobe.

[34] De Biase D, Pennacchietti E (2012). Glutamate decarboxylase-dependent acid resistance in orally acquired bacteria: function, distribution and biomedical implications of the *gadBC* operon. Mol Microbiol.

[35] Laroute V, Yasaro C, Narin W, Mazzoli R, Pessione E, Cocaign-Bousquet M, Loubiere P (1050). GABA production in *Lactococcus lactis* is enhanced by arginine and co-addition of malate. Front Microbiol.

[36] Zhang Y, Song L, Gao Q, Yu SM, Li L, Gao NF (2012). The two-step biotransformation of monosodium glutamate to GABA by *Lactobacillus brevis* growing and resting cells. Appl Microbiol Biotechnol.

[37] Zhao A, Hu X, Pan L, Wang X (2015). Isolation and characterization of a gamma-aminobutyric acid producing strain *Lactobacillus buchneri* WPZ001 that could efficiently utilize xylose and corncob hydrolysate. Appl Microbiol Biotechnol.

[38] Cotter PD, O’Reilly K, Hill C (2001). Role of the glutamate decarboxylase acid resistance system in the survival of *Listeria monocytogenes* LO28 in low pH foods. J Food Prot.

[39] Cotter PD, Gahan CG, Hill C (2001). A glutamate decarboxylase system protects *Listeria monocytogenes* in gastric fluid. Mol Microbiol.

[40] Cotter PD, Ryan S, Gahan CG, Hill C (2005). Presence of GadD1 glutamate decarboxylase in selected *Listeria monocytogenes* strains is associated with an ability to grow at low pH. Appl Environ Microbiol.

[41] Wu Q, Tun HM, Law YS, Khafipour E, Shah NP (2017). Common distribution of *gad* operon in *Lactobacillus brevis* and its GadA contributes to efficient GABA synthesis toward cytosolic near-neutral pH. Front Microbiol.

